# Proteome alterations in human autopsy tissues in relation to time after death

**DOI:** 10.1007/s00018-023-04754-3

**Published:** 2023-04-05

**Authors:** Éva Kocsmár, Marlene Schmid, Miguel Cosenza-Contreras, Ildikó Kocsmár, Melanie Föll, Leah Krey, Bálint András Barta, Gergely Rácz, András Kiss, Martin Werner, Oliver Schilling, Gábor Lotz, Peter Bronsert

**Affiliations:** 1 Department of Pathology, Forensic and Insurance Medicine, Semmelweis University, Budapest, Hungary; 2 Institute of Surgical Pathology, University Medical Center Freiburg, Breisacher Straße 115A, 79106 Freiburg, Germany; 3 Faculty of Medicine, University of Freiburg, Freiburg, Germany; 4 Faculty of Biology, University of Freiburg, Freiburg, Germany; 5 Department of Urology, Semmelweis University, Budapest, Hungary; 6 German Cancer Consortium (DKTK) and German Cancer Research Center (DKFZ), Heidelberg, Germany; 7 Khoury College of Computer Sciences, Northeastern University, Boston, USA; 8 Department of Pathology and Experimental Cancer Research, Semmelweis University, Budapest, Hungary; 9 Biobank Comprehensive Cancer Center Freiburg, University Medical Center, Freiburg, Germany

**Keywords:** Proteomics, Autopsy, Degradation

## Abstract

Protein expression is a primary area of interest for routine histological diagnostics and tissue-based research projects, but the limitations of its post-mortem applicability remain largely unclear. On the other hand, tissue specimens obtained during autopsies can provide unique insight into advanced disease states, especially in cancer research. Therefore, we aimed to identify the maximum post-mortem interval (PMI) which is still suitable for characterizing protein expression patterns, to explore organ-specific differences in protein degradation, and to investigate whether certain proteins follow specific degradation kinetics. Therefore, the proteome of human tissue samples obtained during routine autopsies of deceased patients with accurate PMI (6, 12, 18, 24, 48, 72, 96 h) and without specific diseases that significantly affect tissue preservation, from lungs, kidneys and livers, was analyzed by liquid chromatography–tandem mass spectrometry (LC–MS/MS). For the kidney and liver, significant protein degradation became apparent at 48 h. For the lung, the proteome composition was rather static for up to 48 h and substantial protein degradation was detected only at 72 h suggesting that degradation kinetics appear to be organ specific. More detailed analyses suggested that proteins with similar post-mortem kinetics are not primarily shared in their biological functions. The overrepresentation of protein families with analogous structural motifs in the kidney indicates that structural features may be a common factor in determining similar postmortem stability. Our study demonstrates that a longer post-mortem period may have a significant impact on proteome composition, but sampling within 24 h may be appropriate, as degradation is within acceptable limits even in organs with faster autolysis.

**Supplementary Information:**

The online version contains supplementary material available at 10.1007/s00018-023-04754-3.

## Introduction

For centuries, the autopsy has provided the best quality assurance for clinical diagnostics and further improvement of therapeutic approaches. Currently, numerous research projects, including the most advanced translational multi-omics studies provide deeper insights than ever before into various disease states, especially in the context of cancer [1]. Naturally, tissue specimens and their quality are of pivotal importance for these studies. To our knowledge, such studies are largely based on biopsy samples or surgically resected specimens, often excluding autopsy samples, which however may yield unique insight into advanced disease states.

Leading cancer centers have established Rapid Autopsy Programs (RAPs). These projects aim to obtain the tissue from deceased patients immediately after death to reduce the effect of post-mortem tissue degradation. Several recent studies highlight the usefulness of RAP, e.g. for genomics [2], transciptomics [3, 4] or proteomics [5]. Nevertheless, RAPs remain confined to specialized institutions [6, 7] and it still poses a challenge to harness the possibilities of non-RAP autopsy specimens for translational research. Some authors have sought to keep the post-mortem interval as short as possible by performing core biopsies after death [8]. However, tissue specimens obtained during traditional autopsies are still much more widely available for both cancer and non-cancerous diseases.

Numerous studies highlight the value of autopsy-based samples in biomedical research, with examples covering cancer research [9], improvement of clinical practice and education [10, 11], neuroscience research [12, 13], pathogenesis of COVID-19 disease [14], as well as genetic and tissue-specific gene expression differences in non-cancer individuals [15]. Moreover, autopsy cases can provide large amounts of tissue samples that can be used for immunohistochemical purposes as control tissue from organs from which only biopsy samples are usually available.

Protein expression is a primary area of interest for routine histological diagnostics and tissue-based research projects, but the limitations of its post-mortem applicability remain largely unclear, making it challenging to use for this purpose. It is therefore important to investigate post-mortem temporal kinetics of protein expression patterns to determine the limitations on the relevance of proteomic results based on autopsy samples and their applicability to support more comprehensive conclusions.

In the present study, the proteome of human tissue specimens obtained during a routine autopsy at defined time points has been analyzed via liquid-chromatography tandem mass spectrometry (LC–MS/MS). The objectives of the study presented here were (a) to determine the maximum post-mortem interval (PMI) which is still suitable for the characterization of protein expression patterns; (b) to explore organ-specific differences in protein degradation; and (c) to investigate whether certain proteins follow specific degradation kinetics.

Here, we will demonstrate that the proteomic results can be used to determine the post-mortem time interval as long as protein degradation is sufficiently limited to allow tissue samples to be used for protein-based diagnostic or research purposes.

## Material and methods

### Experimental set-up

The study protocol is in accordance with the Declaration of Helsinki and has been approved by the Ethics Committee of Semmelweis University, Budapest, Hungary (13-1/2015). All autopsies were performed at the Department of Pathology, Forensic and Insurance Medicine, Semmelweis University, Budapest, Hungary. Tissue specimens from the lung, kidney and liver have been derived from patients with predefined post-mortem time interval (6, 12, 18, 24, 48, 72, 96 h) and a lack of specific diseases significantly affecting the preservation of the investigated organs. Corpses were stored at 4 °C between death and autopsy procedure. For each time interval not less than three deceased patients were included.

### Tissue lysis

0.1 mg from each tissue specimen were cut into small pieces, mixed with lysis buffer (200 mM HEPES, pH 7.5 (AppliChem, Germany), 1% acid-labile surfactant (ALS, sodium 3-[(2-methyl-2-undecyl-1,3-dioxolan-4-yl) methoxy]-1-propanesulfonate)), and incubated at 90 °C for 10 min with mild agitation (500 rpm). Subsequently, samples were further homogenized using a PreCellys Lysis Kit (Bertin instruments, USA) for three cycles (each 30 secundum, at 6500 rpm) and sonicated with a Biorupture device for 20 cycles (each 30 secundum ON and 30 secundum OFF). After centrifugation (at 4 °C, for 10 min at max speed), the supernatant was collected, the pH value was adjusted to pH 8.0 and samples were reduced (5 mM TCEP (Sigma Aldrich, Switzerland), 10 min at 95 °C) and alkylated (10 mM iodacetamide (Sigma Aldrich, USA) in darkness at room temperature for 30 min). A double digest with Lysyl Endopeptidase (Wako, Japan) in a ratio of 1:100 for 2 h, at 37 °C, at 500 rpm and trypsin TPCK treated (Worthington, USA) in a ratio of 1:50 overnight at 37 °C, at 500 rpm was performed.

### Sample purification

For the ALS deactivation and acidifying of the peptide solution, incubation was performed with 2% *Trifluoroacetic acid (TFA)* (Thermo Scientific, USA) for 30 min, 37 °C, at 500 rpm. The supernatant was used for purification. The columns [HyperSep SpinTip C18, (Thermo Scientific, USA)] were conditioned by adding elution buffer (0.05% TFA [Thermo Scientific, USA), 68% Acetonitrile (ACN) (Merck, Germany)] and centrifugation. Afterward, the columns were equilibrated by adding wash buffer (0.05% TFA, 8% ACN) followed by a centrifugation step. The peptide solution was loaded twice on the columns. The columns were washed three times with wash solution for 1 min, at 1000×*g* and peptides were eluted with elution buffer for 2 min, at 1000×*g* afterwards. Finally, 2 μg of peptides was dried at 45 °C in a vacuum concentrator and submitted for LC–MS/MS. Preparation of isobarically labeled samples was performed essentially as described previously [16].

### LC–MS/MS measurements

For the mass spectrometry measurements, the dried peptides were re-suspended with 1% formic acid (Fluka, USA). One µg peptides were injected on a Q-Exactive Plus mass spectrometer (Thermo Scientific, San Jose, CA) coupled to an EASY-nLC 1000 UHPLC system (Thermo Scientific). The analytical column was self-packed with silica beads coated with C18 (Reprosil Pur C18-AQ, *d* = 3 Â) (Dr. Maisch HPLC GmbH, Ammerbusch, Germany). For peptide separation, a linear gradient of increasing buffer B [0.1% formic acid and 1% mono ethylene glycol (Sigma-Aldrich, USA) in 80% ACN, (Merck, Germany)] was applied, ranging from 5 to 40% buffer B over the first 90 min and from 40 to 100% buffer B in the subsequent 30 min (120 min separating gradient length). Peptides were analyzed in data-dependent acquisition mode (DDA).

All included samples of each specimen were measured in one run. LC–MS/MS conditions for isobarically labeled samples are as described previously [16].

### Protein identification and quantitation

Raw data were analyzed with MaxQuant (v 1.6.17.0) allowing for zero missed cleavage sites, no variable modifications, carbamidomethylation of cysteines as fixed modification. Furthermore, the digestion Trypsin and label-free quantification options were selected. For the protein quantification label min ratio count was set to 1. Only unique peptides were used for quantification, match-between-run settings were allowed. The Human-EBI-reference database was used for protein identification. (one protein per gene; downloaded from https://www.ebi.ac.uk/ on Jan 11th 2020; iRT sequences added manually, and common contaminants were included by MaxQuant). Analysis of isobarically labelled samples was performed using MSFragger [17, 18] and TMT-Integrator, allowing for zero missed cleavage sites, no variable modifications, carbamidomethylation of cysteines, and TMTpro-labelling of peptide N-termini and lysines as fixed modification.

### Proteomics data processing and analysis

MaxQuant evidence and protein group files were imported into the European Galaxy server (https://usegalaxy.eu) [19] and analyzed with MSstats (v 3.22.0.1) [20, 21] and basic filter tools were applied separately for each tissue. To investigate the degree of time-dependent proteome alterations, the first time point (6 h post-mortem), was used as a reference time point. All other time points were pair-wise compared to the reference time point. Proteins with adjusted *p*-value < 0.05 were considered as significantly fast-degraded (negative fold change) termed as degraded proteins or slow-degraded termed as pseudo-enriched (positive fold change) proteins and exported as tab-separated files.

All further protein identifications, quantitative data processing and statistical analyses were performed using the R programming language (v 4.0.4) running on RStudio (v 1.4.1103-4). For each tissue separately, the protein and peptide identifications, along with their quantitative data, were processed using the MSstats package (v 3.20.3) [20] for summarization of protein quantitation and normalization. Only unique peptides were used for quantitation. Of note, “*Leading Razor Protein”* were used as protein IDs.

Data were log2-transformed before median equalization for normalization. The resulting data were reformatted into a protein abundance matrix and used for further analyses. The distribution of missing values was visually assessed using the *naniar* ^10^package (v 0.6.1) [22]. Random forest-based imputation (*missForest* package v 1.4) [23] was applied as a preprocessing step before data modeling.

The *limma* package (v3.48.1) [24] was used to apply a linear model using time as a continuous variable to detect proteins with either linearly positive or negative associations with time. The p-values arising from these inferential analyses were corrected by multiple testing using the Benjamini–Hochberg method, as applied by *limma*. Proteins showing an adjusted *p*-value smaller than 0.05 after modeling, were considered as associated with time.

To evaluate the general functional annotation of the proteins detected by the linear model, enrichment analyses against Gene Ontology (Biological Processes and Cellular Component and Protein Family) annotation was performed. We used the Bioconductor Gene Ontology (GO.db;) [25] and Human Ensembl (EnsDb.Hsapiens.v86;) [26] annotation databases for the enrichment tests. The analyses consisted of over-representation tests based hypergeometric models as implemented in the *clusterProfiler* package (version 3.16.1) [27]. Proteins with either positive (pseudo-enriched) or negative (depleted) association with Time and a non-adjusted *p*-value < 0.05 after *limma* were used as an input for the tests. The enrichment analysis was performed individually for either pseudo-enriched or depleted proteins within each tissue. All proteins identified in such tissue were used as background, and functional groups presenting an adjusted *p*-value < 0.05 were considered as significantly enriched.

### Data and code availability

Raw spectral files and intermediary identification search results were uploaded to the MassIVE repository (part of the ProteomeXchange consortium). Data can be accessed using the MassIVE accession number MSV000090133 or the ProteomeXchange accession number PXD036059.

The peptide and protein identification files from MaxQuant, together with sample annotation, code for pre-processing, reproducible reports and visualizations for the analysis of protein abundance association with time, are openly accessible via Zenodo and GitHub (Cosenza-Contreras, 2022; https://doi.org/10.5281/zenodo.7007047) [28].

## Results

### Experimental setup and proteome coverage

**Fig. 1:**
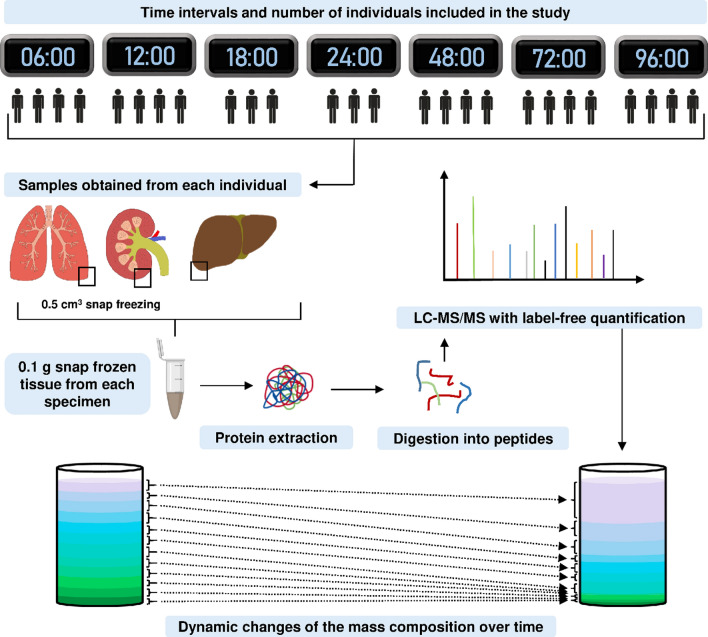
Schematic workflow of the study: For each autopsy time point minimal 3 patients were selected for tissue processing and analysis. Tissue specimens were lysed, proteins were extracted and digested into peptides. The peptides were measured in label-free quantification with LC–MS/MS

The presented prospective study aims to investigate the suitability of autopsy specimens for proteome analyses with such samples having been harvested outside of RAPs, hence representing a more routine setting. The study focuses on three organs, i. e. the kidneys, the liver, and the lungs. The *post-mortem* interval between the time of death and the autopsy was determined as 6 h, 12 h, 18 h, 24 h, 48 h, 72 h, and 96 h. The storage temperature for each corpse was 4 °C. Tissue specimens for each time point was were collected from 3 to 4 individuals (Fig. 1).

General cohort characteristics are shown in Table 1.

**Table 1:** General cohort characteristics

PTI	Age	Gender	M/F ratio
6 h	66	Female	50/50
	56	Male	
	53	Male	
	61	Female	
12 h	62	Female	50/50
	74	Male	
	92	Female	
	37	Male	
18 h	98	Female	66/33
	77	Male	
	62	Male	
24 h	87	Female	33/66
	76	Female	
	68	Male	
48 h	70	Female	25/75
	81	Male	
	78	Female	
	70	Female	
72 h	84	Male	50/50
	88	Male	
	74	Female	
	66	Female	
96 h	79	Male	50/50
	68	Male	
	70	Female	
	84	Female	

*PTI* post-mortem time interval, *M/F* ratio: male/ female

Tissue lysis and protein extraction were based on acid-labile surfactants and bead grinding; hence representing a rather prototypical workflow to ensure wide applicability.

We have employed data-dependent acquisition (DDA) with label-free quantitation (LFQ). Overall, we have identified and quantified the following total protein numbers, when solely considering unique peptides: kidney: 2174 proteins, liver: 2290 proteins, lung: 1785 (Table 2). As with most proteomic studies, not all proteins are simultaneously identified in all samples, resulting in some gaps. Further details on proteome coverage are provided in the subsequent sections.

**Table 2:** The total number of identified unique peptides and proteins by MaxQuant analysis for each organ

Organ	# peptides	# proteins
Kidney	9902	2174
Liver	10,856	2290
Lung	8371	1785

The proteome coverage of this study is somewhat limited. The instrumental setup is well suited to identify > 3500 proteins from cell-line derived samples (not shown). The decreased proteome coverage for the tissue-based samples might reflect sample-intrinsic properties, e.g. a less balanced proteome with more overshadowing components. Consequently, the present study is limited to post-mortem-alterations of abundant proteins, while the impact on less abundant proteins and post-translational modifications remains beyond its scope.

By using MaxQuant and its LFQ algorithm, we utilize an approach that is robust and well-established [29]. The instrument and measurement setup have been successfully used in previous studies employing label-free quantitative proteomics [30–32]. In preparation for using label-free quantitative proteomics with our setup, we have validated technical reproducibility. Consecutive LC–MS/MS measurements of the same sample (“technical replicates”) achieved correlation coefficients > 0.98 for the LFQ protein intensities (not shown). Moreover, we employed spiked-in retention normalization peptides (iRT) as a quality control measure [33]. In the kidney data, nine peptides of the iRT panel were consistently identified and quantified; in the liver and lung data, this was the case for ten iRT peptides.

### Alteration of proteome coverage post mortem

**Fig. 2:**
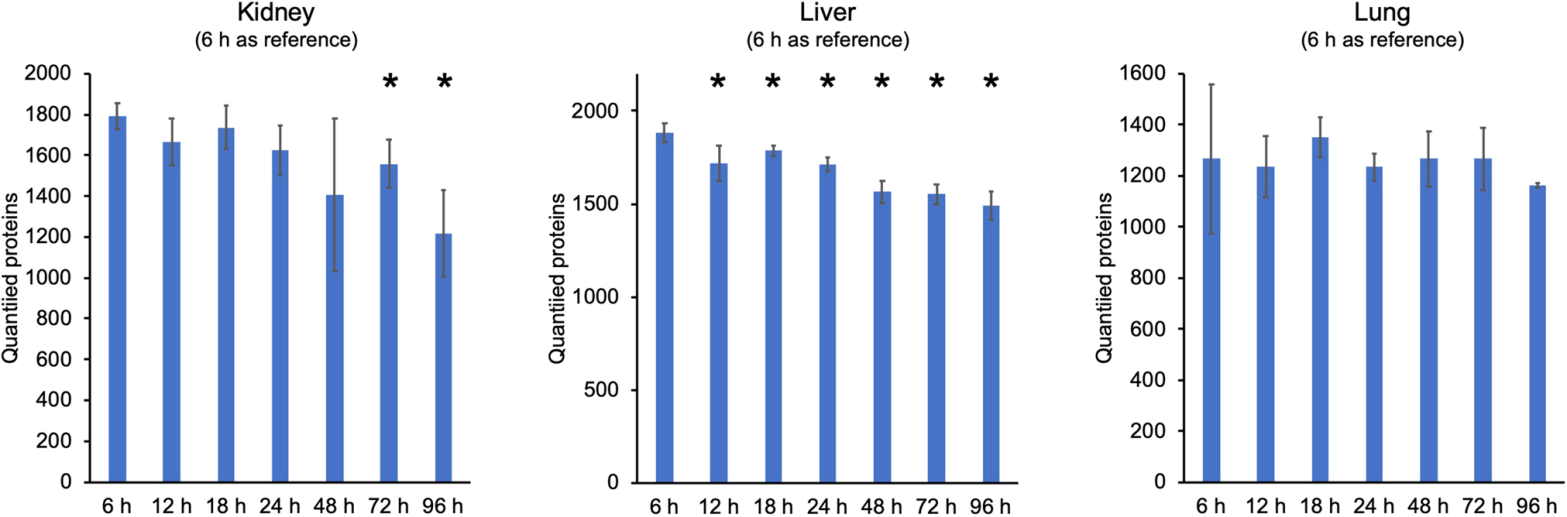
Proteome coverage per organ and time—point; three–four biological replicates (see Materials and Methods) per organ and time point; single LC–MS/MS run per sample. 6 h post mortem was set as the reference time point and alterations of proteome coverage were probed by a two-tailed Student’s t-test. A star denotes significantly (*p* < 0.05; two-tailed t-test, 6 h post mortem was set as the reference) affected proteome coverage

As initially outlined, the primary aim of the presented study is to investigate at which time point *post-mortem,* autopsy material has decayed to such an extent that it is no longer amenable to proteomic analysis. To this end, corpses were stored at 4 °C and autopsy time points of 6 h, 12 h, 18 h, 24 h, 48 h, 72 h, and 96 h after the patients’ demise were chosen to collect autopsy specimens of kidney, liver, and lung tissue; with n = 3 or 4 for each organ at each time point. The proteome coverage (three replicates per organ and time-point; a single LC–MS/MS runs per sample) per organ and time point is shown in Fig. 2. For the kidneys and the liver, we observe a slight decline of protein ID numbers upon prolonged time intervals. When setting 6 h *post-mortem* as the reference time point, a mild decrease in protein IDs emerges as being significant (*p* < 0.05; two-tailed *t*-test) at 72 h and 96 h for kidney tissue and at each time point for the liver. For the lung tissue, the protein coverage appears rather constant. In the case of the liver, proteome coverage at 24 h still reaches > 90% of the proteome coverage at the reference time point of 6 h. We conclude that for kidney, lung, and—to a lesser extent—liver tissues, autopsy sampling at 24 h *post-mortem* allows for sufficient proteome coverage.

### Alteration of proteome composition post mortem

**Fig. 3:**
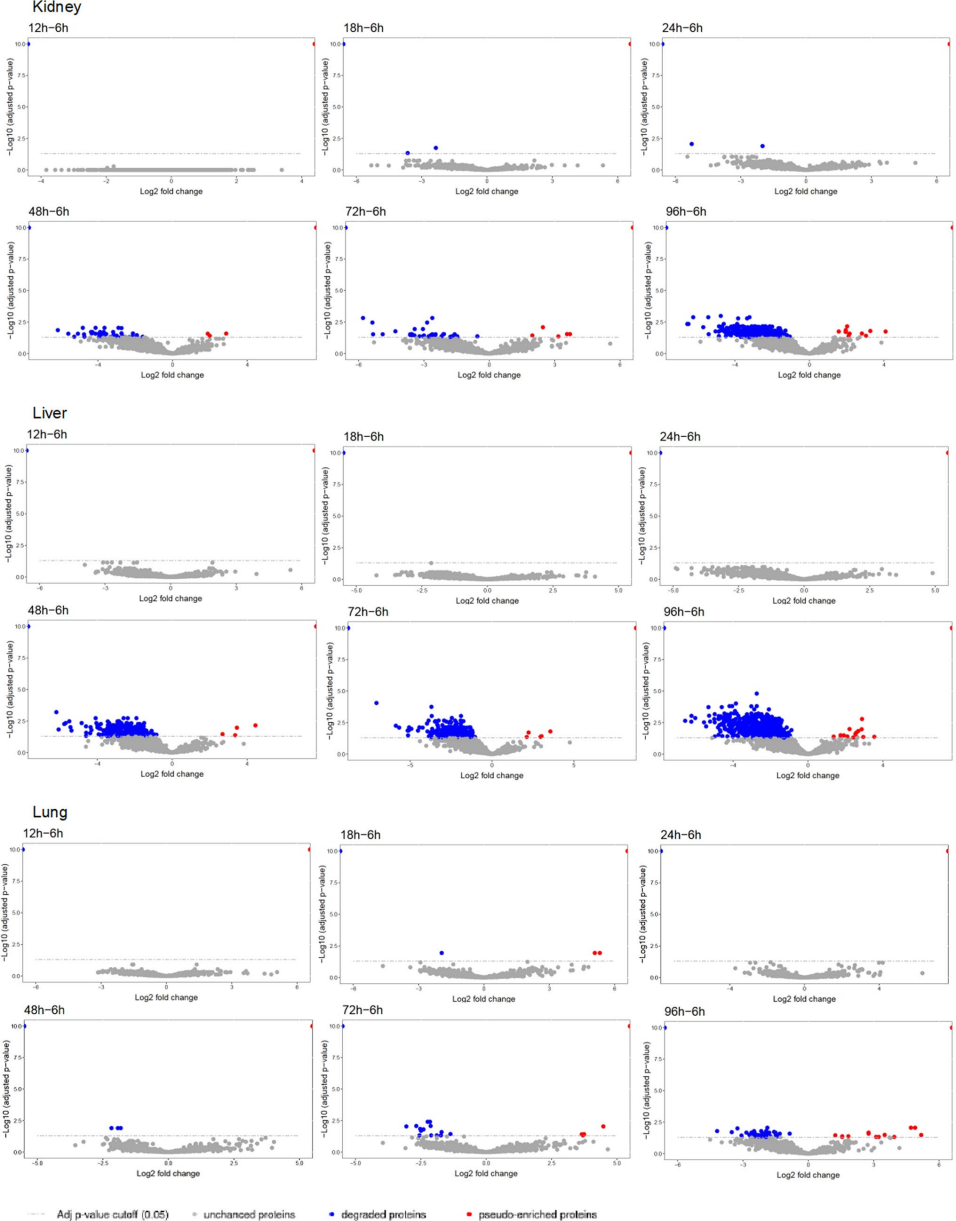
Limma comparison between 6 h (reference time point) and subsequent time points for kidney, liver and lung. On the left side of the volcano plot, degraded proteins are disposed. On the right side, pseudo-enriched proteins are arranged. Data points in blue show the statistically significant degraded proteins (adjusted *p*-value ≤ 0.5). Those in red show statistically significant pseudo-enriched proteins over time (adjusted *p*-value ≤ 0.05). The grey data points show no statistically significant relevance in degraded and pseudo-enriched proteins

Next, we set out to identify the quantity of significantly degraded proteins per organ and the optimal time point for *post-mortem* sampling. We have chosen 6 h sampling as the reference time point and employed linear models of microarray analysis (limma) [24] as implemented in MSStats [20]. Of note, the same amount of proteome per sample (300 ng) has been injected into the LC–MS/MS setup. Hence, our analysis captures alterations of proteome composition: while rapidly degrading proteins vanish, more stable proteins become more prevalent within the mixture, leading to a pseudo-enrichment despite the absence of widespread protein synthesis *post-mortem*. The pair-wise comparisons to the 6 h reference time point are shown in Fig. 3. Proteins with an adjusted *p*-value < 0.05 have been considered as either being depleted by degradation or as being more stable and hence being pseudo-enriched.

**Fig. 4:**
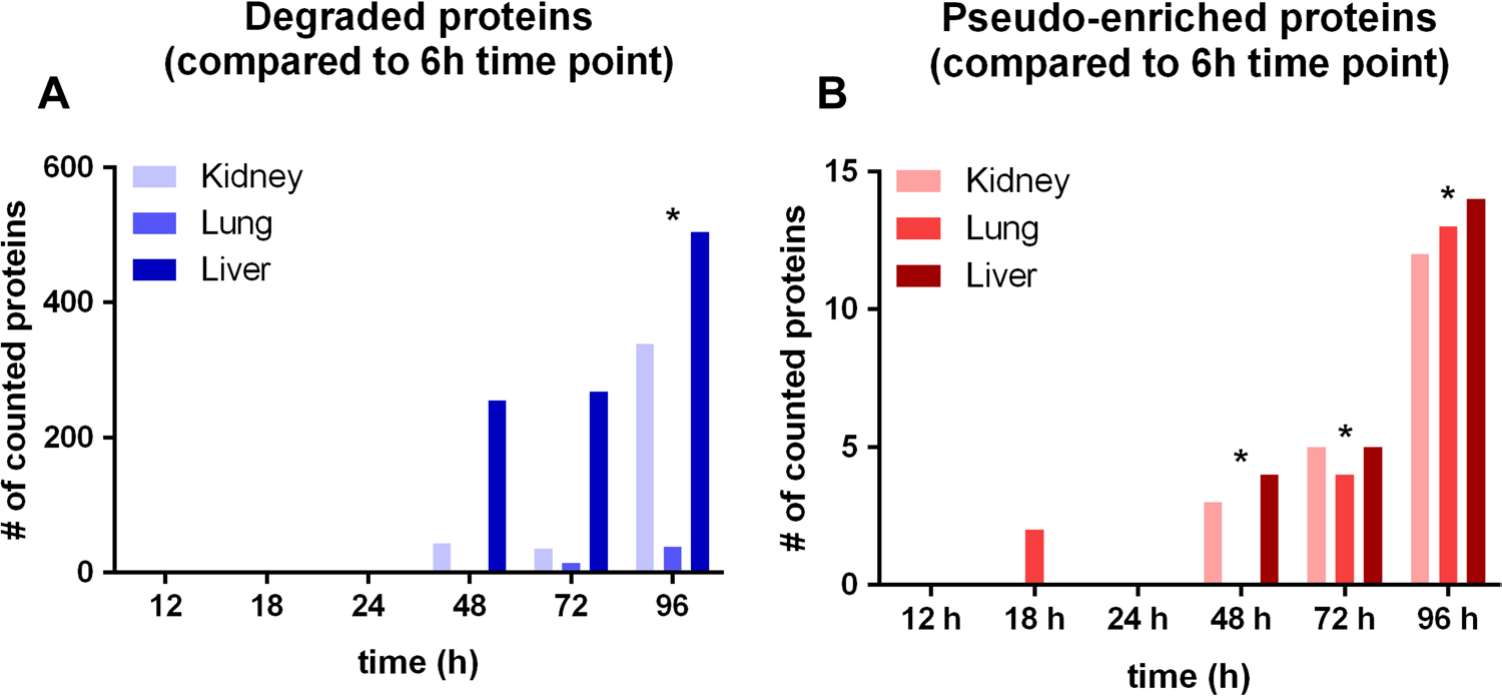
Numbers of the degraded proteins over autopsy time points compared to 6 h (**A**) and numbers of pseudo-enriched proteins over autopsy time points compared to 6 h (**B**). Statistically significant changes (black asterisk) occur after 24 h after patients’ demise with an adjusted *p*-value ≤ 0.5

For the kidneys and the liver, we have noticed very few significant proteome alterations at 18 h and 24 h while substantial and significant protein degradation becomes apparent at 48 h. For the lungs, substantial protein degradation only becomes apparent at 72 h and the proteome composition appears to be rather static for up to 48 h. The quantities of significantly degraded or pseudo-enriched proteins are summarized in Fig. 4. We conclude that for the kidneys and the liver, autopsy sampling for up to 24 h allows for quantitative proteome studies that are likely to be representative of the proteome biology at the time of death. For the lungs, this time point is up to 48 h. Of note, this approach focuses on abundance proteomics and does not include post-translational modifications which may present more dynamic alterations.

As an additional overview of the proteomic data, we performed principal component analysis (PCA) for proteins that were ubiquitously quantified in all samples for either kidney, lung, or liver using MetaboAnalyst [34] (Supplementary Fig. 1.). Missing values present a complication for approaches such as PCA or hierarchical clustering and typically require an imputation step. In our study, we observe a partially time-dependent increase in missingness. We refrained from imputation for PCA and focused on proteins that were ubiquitously quantified in all samples per organ. A clear segregation of samples with different (short or prolonged) post-mortem times does not become apparent by PCA.

### Identifying proteins with defined kinetics of degradation and pseudo-enrichment

In addition to the previous statistical analysis, we have sought to identify proteins that present a defined (i.e. statistically significant) degradation or pseudo-enrichment during storage for up to 96 h at 4 °C. To this end, we have employed limma statistics with time as a continuous variable. In kidney tissue 402 (Supplementary Fig. 2A), in liver tissue 235 (Supplementary Fig. 2B), and in lung tissue ten (Supplementary Fig. 2C) proteins have been identified as reaching significance (adjusted *p*-value < 0.05) for a linear model with time as a continuous variable.

Of note, linear models—as used in the present study—can only grasp those proteins whose degradation (or pseudo-enrichment) kinetics fits the proposed statistical model. We show this approach as a means to demonstrate that there is a systematic trends in post-mortem protein degradation. We do not imply that protein degradation generally follows a linear trend.

The comparison of the identified proteins has demonstrated that 43 proteins of kidney and liver tissues have the same linear behavior. Six proteins have opposite behavior in each organ (Supplementary Tables 1–3). Within the kidney specimens O00468 (agrin), Q04917 (14–3-3 protein eta), and Q92499 (ATP-dependent RNA helicase DDX1) have shown pseudo-enrichment behavior (positive linear correlation) whereas in liver samples they exhibited degradation behavior (negative linear correlation). The proteins O43493, P10636 and P21796 have demonstrated degradation behavior (negative linear correlation) in kidney specimens and pseudo-enrichment behavior (positive linear correlation) in liver specimens. A pairwise comparison of kidney and lung tissues revealed four proteins with pseudo-enrichment behavior and one protein with degradation behavior. When liver and lung samples were compared, two identical proteins were identified that showed the same linear characteristic behavior (one of them decreased, the other one pseudo-enriched). (Supplementary Tables 1–3). We conclude that degradation kinetics appear to be organ specific.

For each tissue specimens (kidney, liver and lung) the top proteins with the lowest adjusted *p*-value (≤ 0.05) have been selected and further analyses of emerging behavior patterns of their abundance over time (6 –96 h) after patients’ demise have been conducted.

**Fig. 5:**
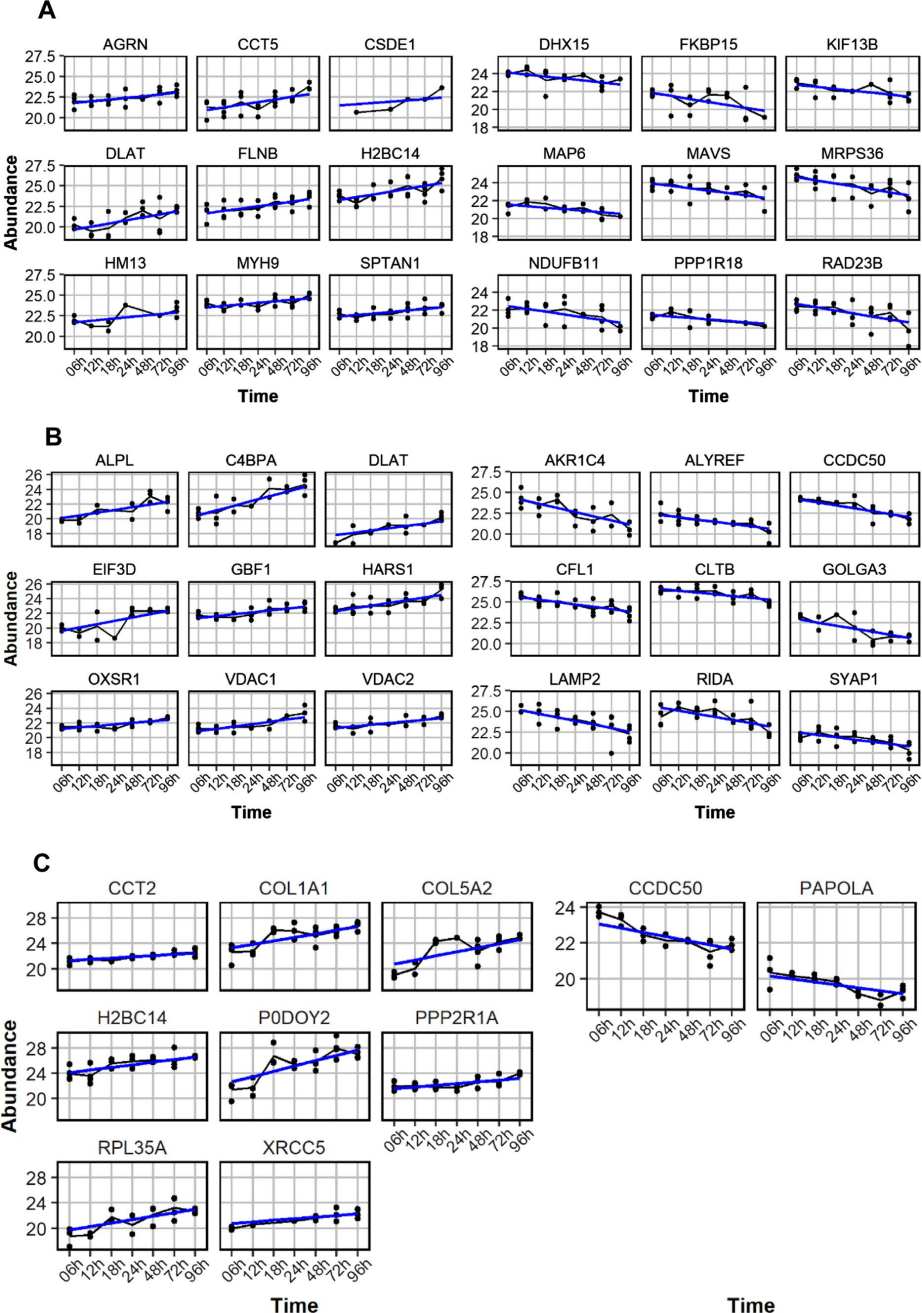
For exemplary visualization the Top 9 protein abundance with a positive and negative correlation over time of kidney (**A**), liver (**B**) and lung (**C**) are represented. The proteins had the lowest adjusted *p*-value ≤ 0.05. Selected autopsy time points were 6 h, 12 h, 18 h, 24 h, 48 h and 96 h. The analyzed proteins are from the previous analysis of degrading and pseudo-enriched proteins in correlation over time

All degraded and pseudo-enriched proteins show similar behavior over time shown as an example in the top 9 proteins (Fig. 5) of each specimen.

### Organ-Specific gene ontological patterns of degradation and pseudo–enrichment in kidney, liver, and lung tissues

**Fig. 6:**
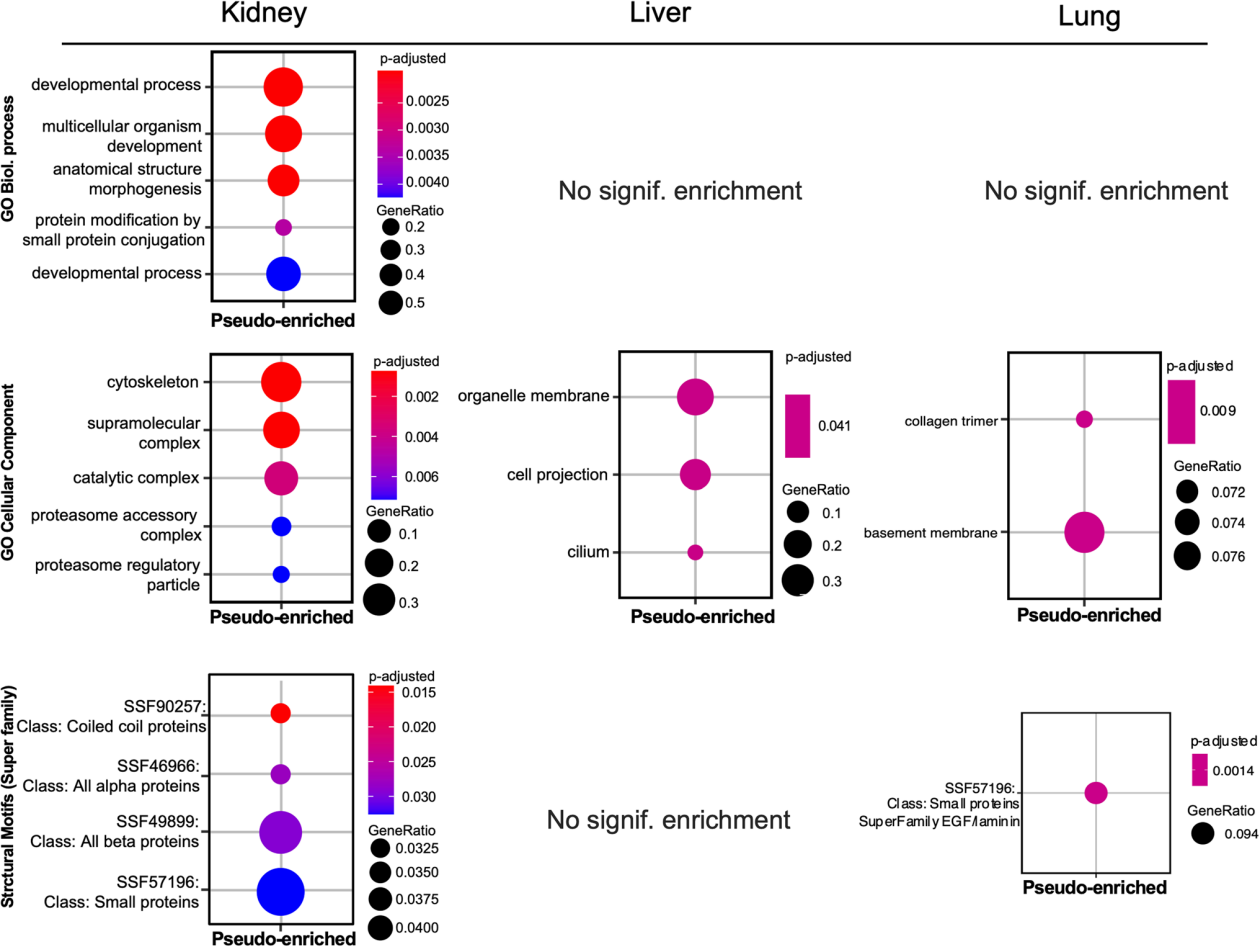
Over-representation analysis results for kidney, liver and lung. Pseudo-enriched proteins per tissue were selected from the *limma* analysis results based on a non-adjusted p-value < 0.05. GeneRatio represents the fraction of pseudo-enriched proteins associated to the tested annotation term

Based on the collection of proteins identified by pseudo-enrichment and degradation kinetics, an enrichment analysis has been applied to investigate their distribution by cellular components (Gene Ontology database) and structural motifs (Protein Family) as well as their role in biological processes. Only pseudo-enriched (i.e. stable) proteins have been found to show an over-representation of annotated biological terms. Regarding kidney tissue, these proteins are over-represented in terms associated with developmental processes (GO:BP), cytoskeletal proteins, and supramolecular complexes such as proteasomal units (GO:CC). Pseudo-enrichment of kidney proteins with various structural motifs has also been identified, with the most significant enrichment of coiled-coil proteins. Pseudo-enriched proteins in liver tissue have been associated with organelle membrane and cell projection cellular components. In lung tissue, pseudo-enriched proteins show significant enrichment of basement membrane and collagen trimmer cellular components, together with structural motifs associated with the EGF/laminin domain. In summary, no similar pattern of over-representation of biological motifs for pseudo-enriched or degraded proteins has been found among the studied tissues (Fig. 6).

### Corroboration by isobaric labelling—based quantitative proteomics

**Fig. 7:**
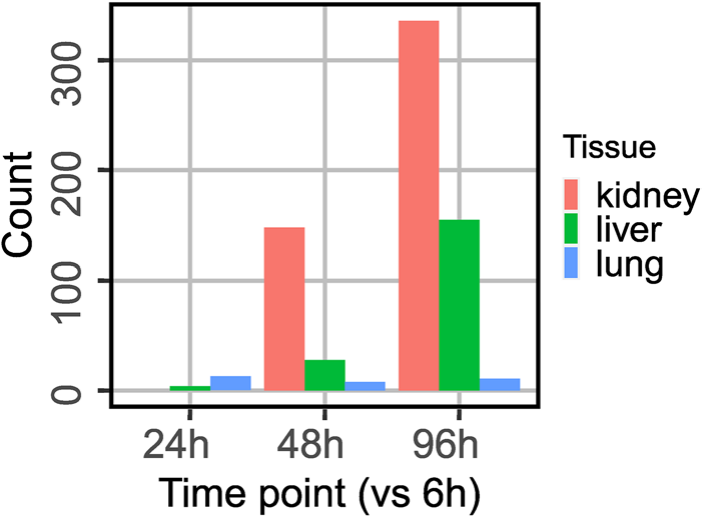
Numbers of the degraded proteins per autopsy time points compared to 6 h as determined by isobarically labelled data

Isobaric labelling together with prefractionation provides a strategy for enhanced coverage in quantitative proteomics. We probed the samples representing 6 h, 24 h, 48 h, 96 h *post-mortem* using 16-plex isobaric labelling with separate labelling pools for the kidney, liver, and lung. These samples also represent different sampling areas as compared to the LFQ samples. Scarcity of material reduced the number of fractions yielded by prefractionation to less than six. This approach enhanced proteome coverage to 3818 proteins for the kidney, 3709 proteins for liver, and 3453 proteins for the lung tissue. As described for the LFQ data, we used limma statistics to determine the numbers of the degraded proteins per post-mortem time points compared to 6 h. In good agreement with the LFQ data, kidney stands out as the most affected tissue while the lung is comparably stable (Fig. 7).

## Discussion

The main objectives of our study have been to investigate the process of protein degradation after death using quantitative mass spectrometry and determine the time interval after death beyond which autolytic processes render tissue samples unsuitable for protein expression studies.

In recent years, an autopsy has become a focus of interest for several reasons. One reason is that the currently available bioinformatics databases are mainly based on data obtained from surgical resection specimens. Due to the fact that most tumors are diagnosed at an inoperable high/advanced tumor stage the number of available tissue specimens is limited to core needle biopsies.

Accordingly, rapid autopsy programs were primarily designed to obtain tissue samples from patients with advanced tumor stage. The longer the post-mortem interval, the greater the chance of degradation of the macromolecules of interest, so these programs describe the procedures specifically designed to retrieve fresh tissue from patients within a short time after death. [7] Depending on the preferences and regulations of a given centre and the specific aspects of the individual case (pre-mortem conditions: e.g. inflammatory conditions, especially septic processes that may accelerate postmortem tissue degradation, or agonal factors such as prolonged hypoxia due to respiratory and/or cardiac failure, microcirculatory disturbance, shock, but febrile conditions or, conversely, death due to hypothermia may also affect postmortem decay; post-mortem conditions (e.g. external temperature, humidity)), the maximum acceptable post-mortem interval varies between 0.5 and 23 h [7].

As rapid autopsy programs have focused primarily on creating conditions that satisfy RNA- and DNA-based molecular approaches, we have considered it important to clarify the issue of post-mortem interval length from a proteomic perspective. As the rate of degradation of macromolecules can vary greatly between different organs, tissue types and subtypes, and between different molecules, we first aimed to determine the kinetics of degradation in various organs using proteomic methods.

Although RAP programs are becoming more widely available, it is mainly researchers who benefit from them. However, the diagnostic and pathogenetic questions of diseases that can be investigated by post-mortem sampling may be of interest to a much wider audience, and for the time being, autopsy case samples from routine pathological practice are available to answer these questions. Therefore, the aim of our study was to investigate the potential of protein expression analysis of autopsy tissue samples handled under routine conditions. To simulate these routine diagnostic circumstances, we have selected tissues of organs most commonly examined by post-mortem histology in our autopsy practice, and where the decomposition is neither too fast nor too slow, namely the lungs, the liver and the kidneys.

Basically, we explored the issue through the following two approaches: a) post-mortem alteration of proteome coverage, b) post-mortem alteration of proteome composition.

Recently, more and more innovative approaches to protein identification and quantification are becoming available that can provide deeper information, e.g. aptamers that also consider the structural features of proteins in their identification [35–37]. We have used LC–MS/MS because it has a solid knowledge base due to its wide use and compatibility with different sample types, including formalin fixation and paraffin embedding. LC–MS/MS technology is not primarily suited for spatially resolved probing of proteomic heterogeneity or for demonstrating proteome changes in different organ regions, and thus no attempt is made to extend it in this direction in the present study.

Regarding the proteome coverage, a slight decline was found by the time for kidney and liver tissue, however, it was rather constant up to 72 h in the case of lung tissue. As the most significant changes were found in kidney and liver tissues after 24 h, and proteome coverage still reached > 90% of the 6-h value at 24 h, we conclude that 24 h post-mortem may be the maximum acceptable time interval beyond which samples are not suitable for quantitative protein expression studies.

The post-mortem alteration of proteome composition was analyzed in our cohort by testing the same amount of total proteins at different post-mortem time points by LC–MS/MS. In this approach, candidates with decreasing amounts over time represent the degrading proteins, while proteins with pseudo-enrichment kinetics correspond to the (more or less) stable ones. Our proteome composition results also supported that substantial and significant protein degradation becomes apparent after 24 h in liver and kidney tissues and after 48 h in the lung tissue. Consequently, a longer post-mortem period may have a significant impact on the proteome composition (differential degradation), but sampling within 24 h may be appropriate, as degradation is within acceptable limits even in organs with faster autolysis.

By analyzing organ-specific gene ontological patterns and identifying specific proteins that show statistically significant degradation or pseudo-enrichment, we can gain deeper insights into these processes. This may be of interest in terms of which proteins or protein families belong to the more degradable or stable subgroups. By identifying such patterns and revealing their similarities and differences between different organs, we can gain a better understanding of the degradation patterns that are well-known from macroscopic and microscopic autopsy pathology, e.g. differential and organ-specific autolysis. Moreover, by further refinement, such as microdissection sampling, it may be possible to answer later why, for example, in the renal cortex the proximal tubules are affected first, followed by the distal tubules and glomeruli.

Thus, we have tried to classify these proteins according to the biological processes, cellular components (Gene Ontology database) and structural motifs (Protein Family). However, based on the databases listed, it was not possible to classify proteins that are significantly degradable in any of the three organs. Furthermore, it was not possible to classify liver proteins showing pseudo-enrichment according to these databases. However, enrichment analysis based on the GO Cellular Components database indicated an enrichment of liver proteins localized to organelle membranes, cell projections, and cilia. In the kidney, which is known to have very quickly degrading tissue components, we were able to find numerous pseudo-enriched proteins classified according to the biological processes, cellular components or structural motifs. Interestingly, pseudo-enriched kidney proteins were involved mainly in developmental and morphogenesis processes. More interestingly, classification by cellular components showed pseudo-enrichment of cytoskeletal and supramolecular complex proteins in the kidney tissue, including actin and cortical cytoskeleton proteins, intermediate filaments and microtubules, collagen network, etc., as well as catalytic proteins such as different metabolic and degrading enzymes (endonuclease, peptidases), and proteasome-related proteins.

Since hypoxia and degradation at the cellular-subcellular level first affect the integrity of membranes and thus cause degradation of membrane-associated proteins and disorganization of membrane-bound subcellular compartments, the relative conservation and pseudo-enrichment of cytoskeletal proteins is in line with previous observations [38]. Peptidases and the proteasome (a large complex that catalyzes the degradation of proteins) are factors involved in the degradation of proteins and may therefore play a key role in autolytic processes, therefore their pseudo-enrichment may explain the rapid decomposition of some renal tissue components. Classification by structural motifs (protein family) also revealed an enrichment of structural proteins such as cytoskeletal (non-muscle cytoplasmic myosin, spectrin) and basal lamina (laminin) proteins. These results suggest that proteins with similar post-mortem kinetics are not primarily shared in their biological functions but are more likely to be similar in their sensitivity to autolysis. The overrepresentation of protein families with analogous structural motifs in the kidney indicates that structural features may be another common factor in determining similar postmortem stability.

Post-mortem proteomics is an emerging field, a similar potential application of this could be the determination of PMI. In the field of forensic pathology, some authors have attempted to estimate the time after death from the postmortem degradation of skeletal muscle proteins [39, 40]. Our results could help to potentially extend this to the examination of tissue samples from internal organs; however, the present study does not investigate the impact of different storage temperatures, which is highly relevant in the field of forensics. Similar studies on post-mortem proteome alterations have been performed in animal tissues [39, 41, 42]. A prime example is the post-mortem aging of beef, which has been the focus of several proteomic studies [43, 44]. However, the primary interest of these has been in muscle proteins rather than proteins of organs such as the kidneys or the liver. Moreover, several studies have investigated the effect of PMI on DNA/RNA degradation kinetics, or DNA/RNA preservation, in human samples. However, despite a careful literature search, we are not aware of any proteomic investigation similar to the study we have performed. Several studies present disease-specific proteome alterations based on autopsy samples, but these studies have not evaluated the impact of PMI on proteome integrity [5, 8, 45].

Some further limitations of the presented study also have to be mentioned. Firstly, the sample size per time-point is limiting. Another major point is, that the tissue specimens withdrawn from the autopsy at different time points are not from one, but from different deceased persons. Thus, it is possible that, although we have attempted to establish and adhere to standard conditions of adequacy for sampling, as we are dealing with different individuals, not only PMI but also individual variation may influence the differences in samples from different time points. It is also important to emphasize that our results only reflect the degradation of normal tissue components, not the neoplastic lesions, which may exhibit a more accelerated degradation process (due to tissue hypoxia and necrosis). Therefore, further studies will also be needed to validate the appropriateness of the 24-h maximum post-mortem period for tumor tissue sampling. Since we have found shorter acceptable PMI for the liver and kidney than for lung in terms of both proteome coverage and proteome composition. These results raise the question of whether the differences may be due to the anatomical location of the organs in addition to their different composition. In fact, the proximity of the colon to these two organs provides an opportunity for the transposition of bowel bacteria [46] and thus an additional heterolytic factor increasing tissue decomposition. A previous study from Hurtado et al. has shown a significant increase in bacterial contamination—especially with the *Enterobacteriaceae* and *Pseudomonas* spp.- after 24 h postmortem [46, 47]. A further limiting factor may be that in some animal studies, mRNA expression has been found to be active in certain cell types up to 48 h post-mortem [48]. This could theoretically add to the pseudo-enrichment of proteins by increasing it with additional (real) protein production, but no data on post-mortem translational activity are available, we hypothesise that it may not significantly affect our results—if it even exists.

In conclusion, using proteomics as a clock instead of the post-mortem interval, we can see that this clock ticks at different rates in different tissues. Our post-mortem proteomic approach may pave the way to determine the time interval after death until the overall preservation of proteins allows the use of tissue samples for protein-based diagnostics or research, and to determine the same for specific proteins, protein groups or protein families to be tested.

## Supplementary Information

Below is the link to the electronic supplementary material.

Supplementary file1 (DOCX 986 KB)

## Data Availability

Raw spectral files and intermediary identification search results were uploaded to the MassIVE repository (part of the ProteomeXchange consortium). Data can be accessed using the MassIVE accession number MSV000090133 or the ProteomeXchange accession number PXD036059. (During reviewing process the data can be accessed using the following credentials: FTP address -ftp://MSV000090133@massive.ucsd.edu; username: MSV000090133; password: necrodegradomics). The peptide and protein identification files from MaxQuant, together with sample annotation, code for pre-processing, reproducible reports and visualizations for the analysis of protein abundance association with time, are openly accessible via Zenodo and GitHub (Cosenza-Contreras, 2022; https://doi.org/10.5281/zenodo.7007047) [28].
