# Supplementary material for: Proteome alterations in human autopsy tissues in relation to time after death
Source: Cell Mol Life Sci. 2023 Apr 5;80(5):117. doi: 10.1007/s00018-023-04754-3 (PMC10075177; doi:10.1007/s00018-023-04754-3)
Supplement: Supplementary file 1 — Supplementary file1 (DOCX 986 KB) [file 18_2023_4754_MOESM1_ESM.docx]

**Supplementary Figure 1**

A)


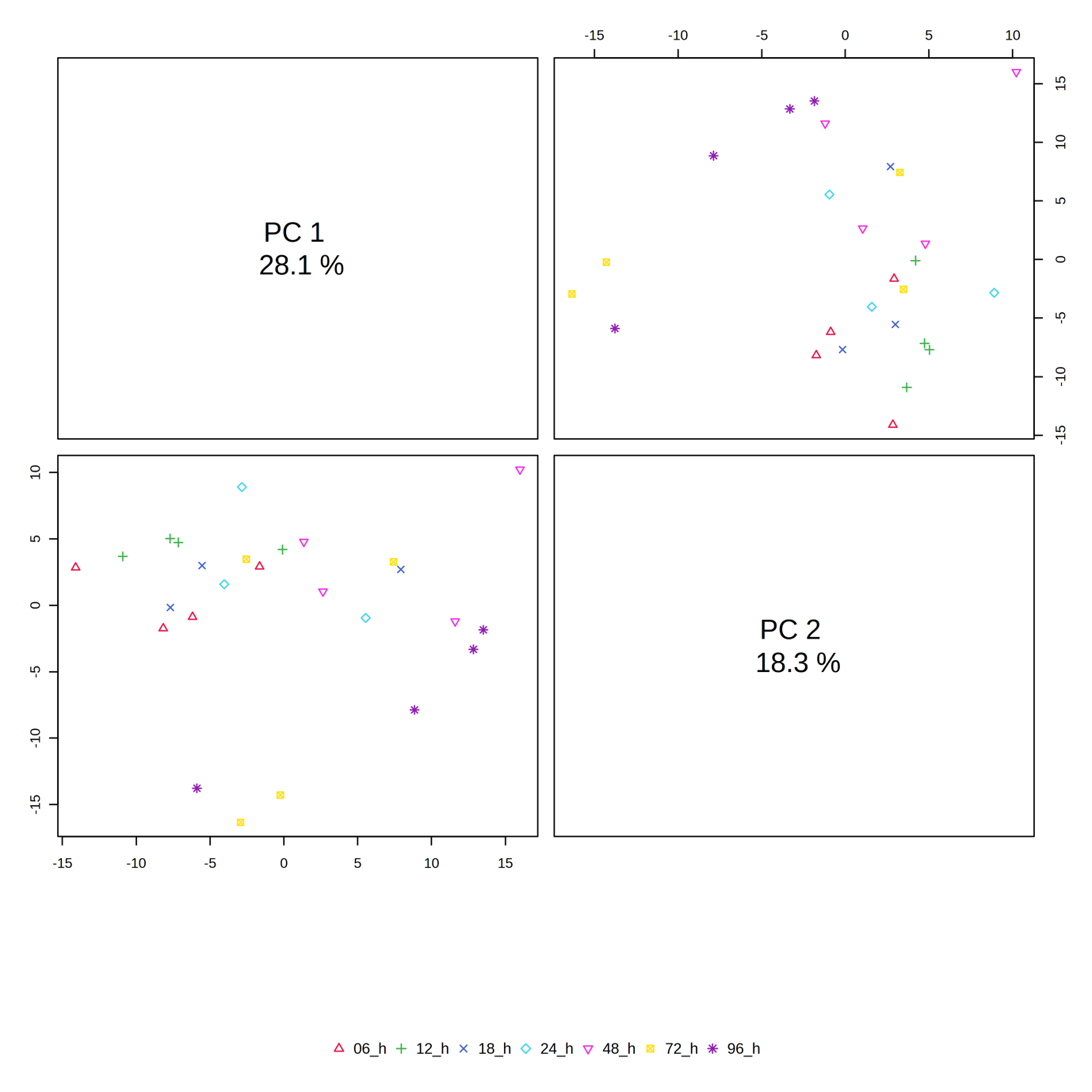

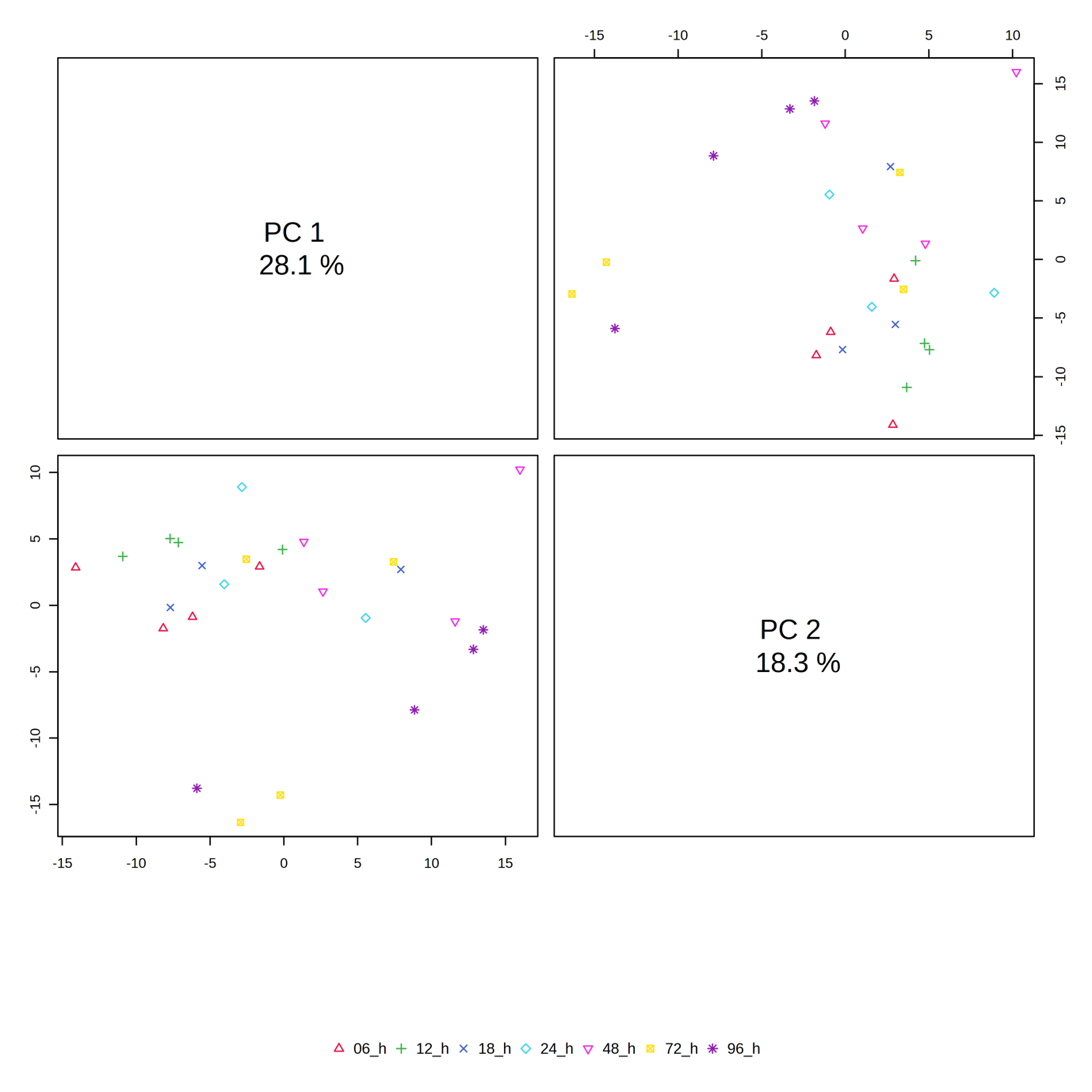


B)


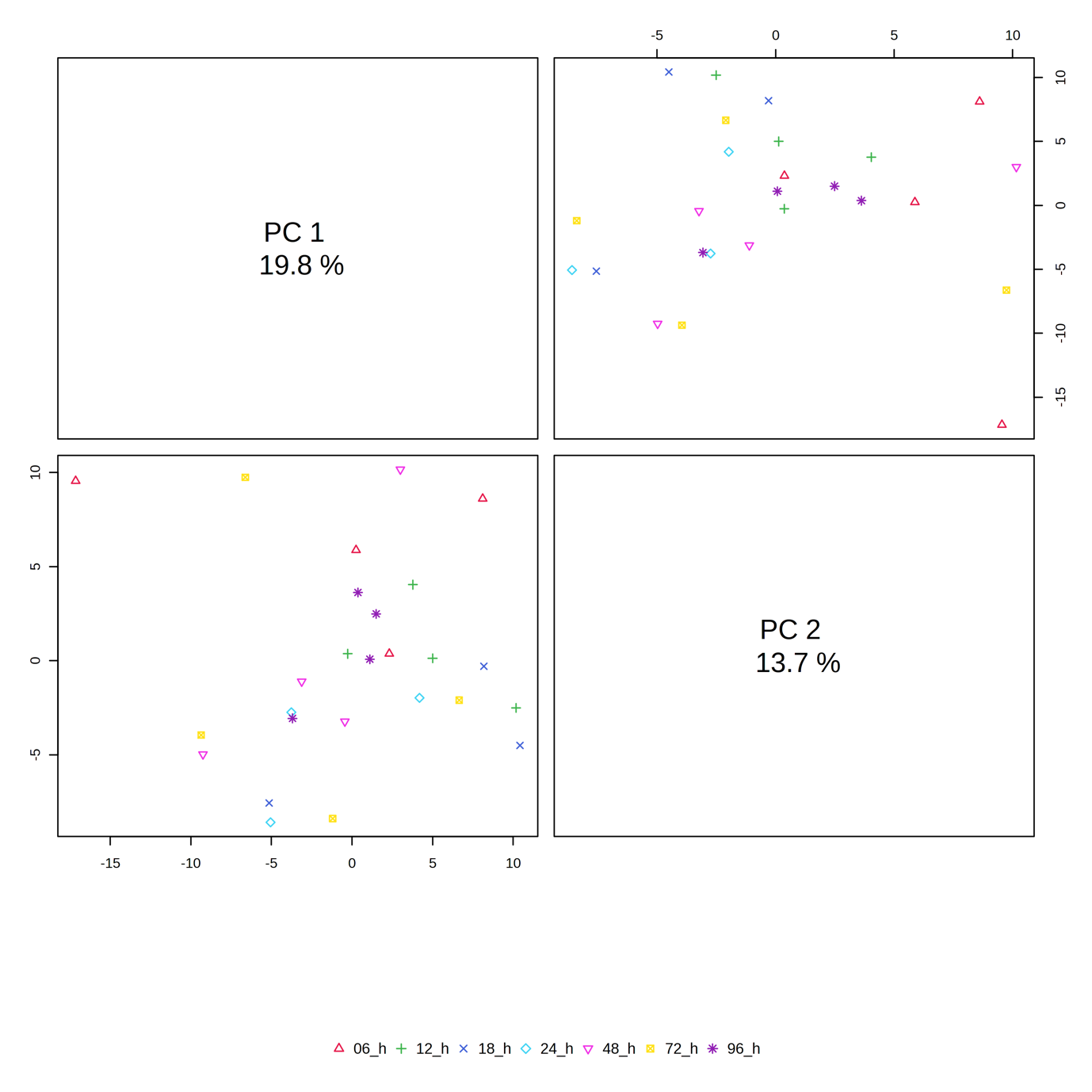

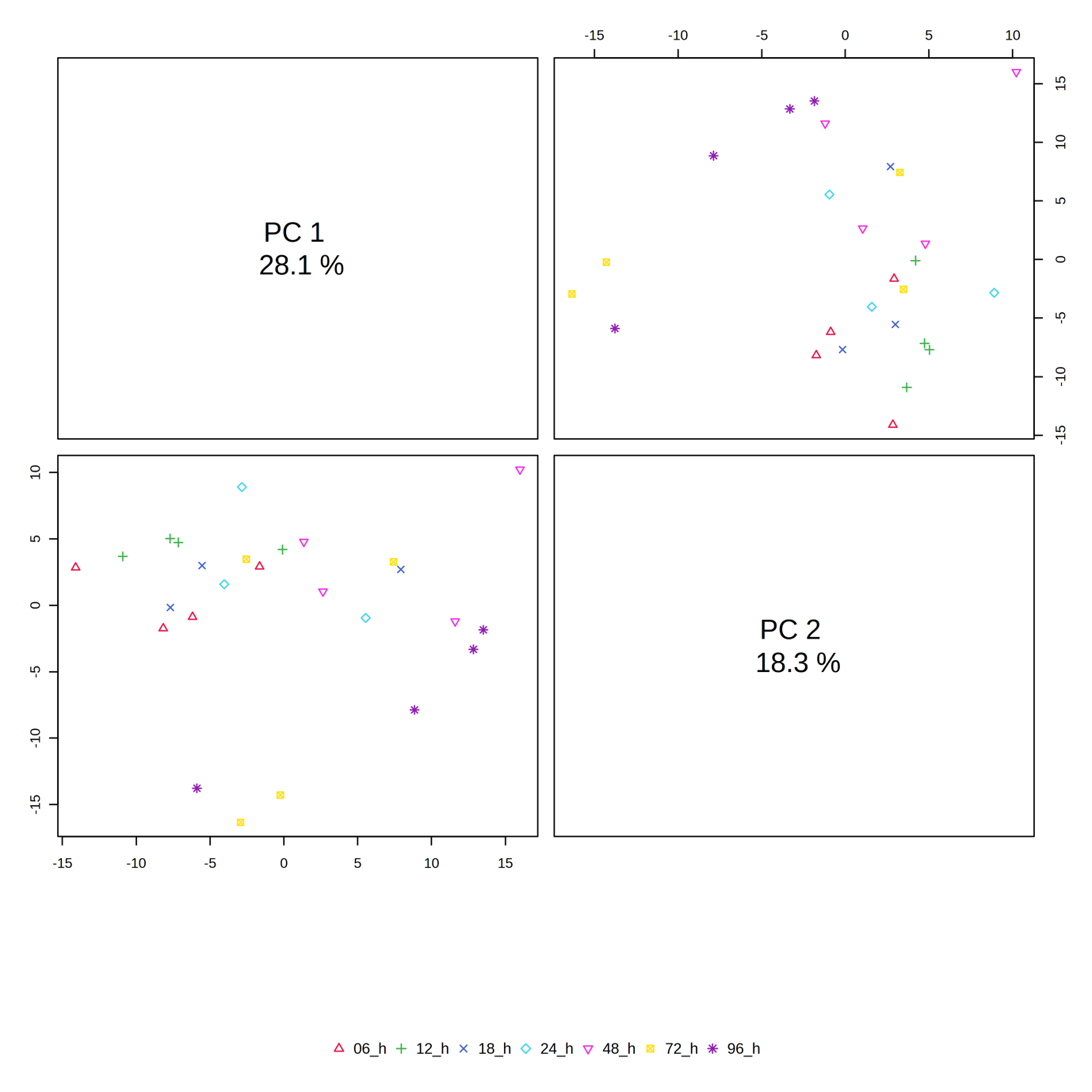


C)


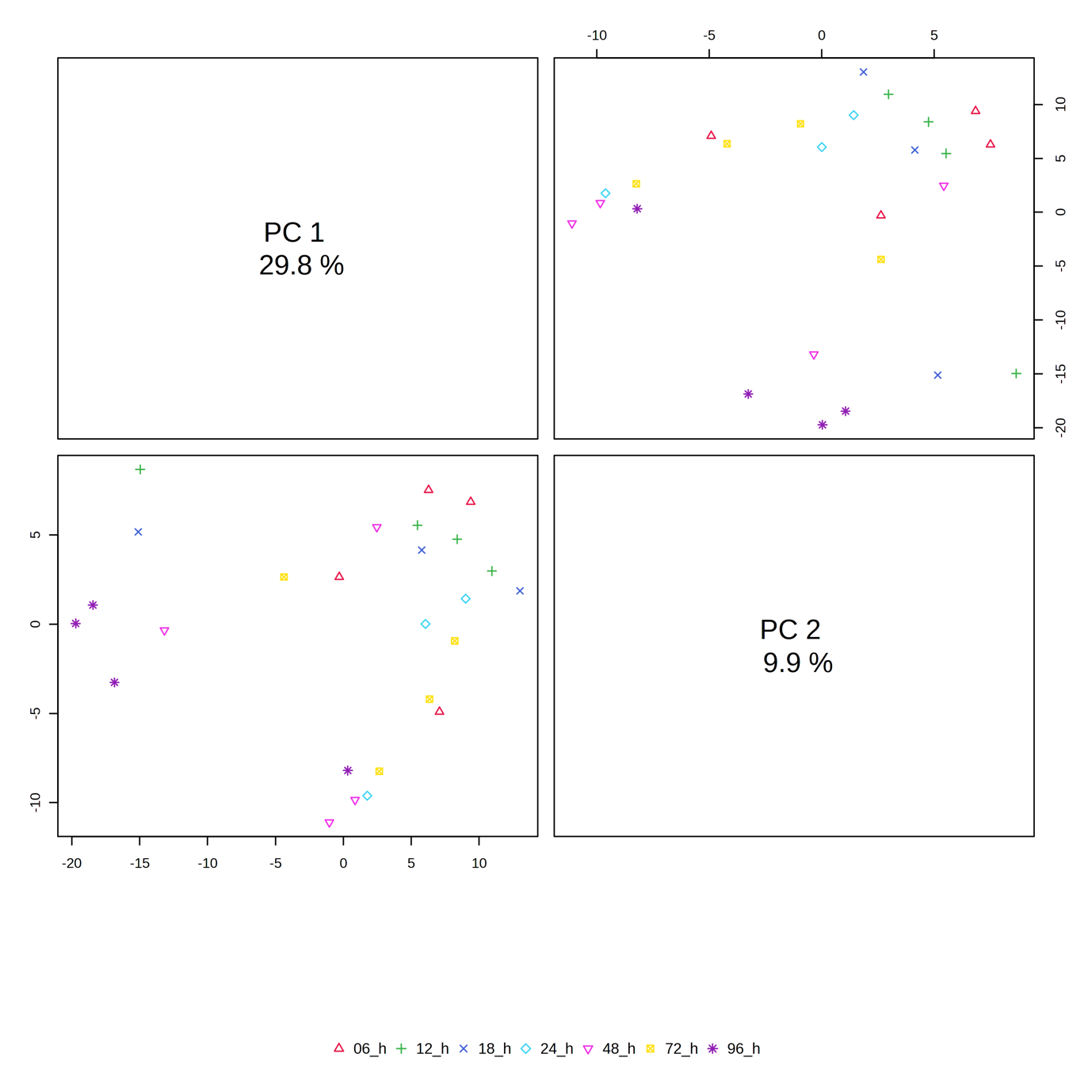

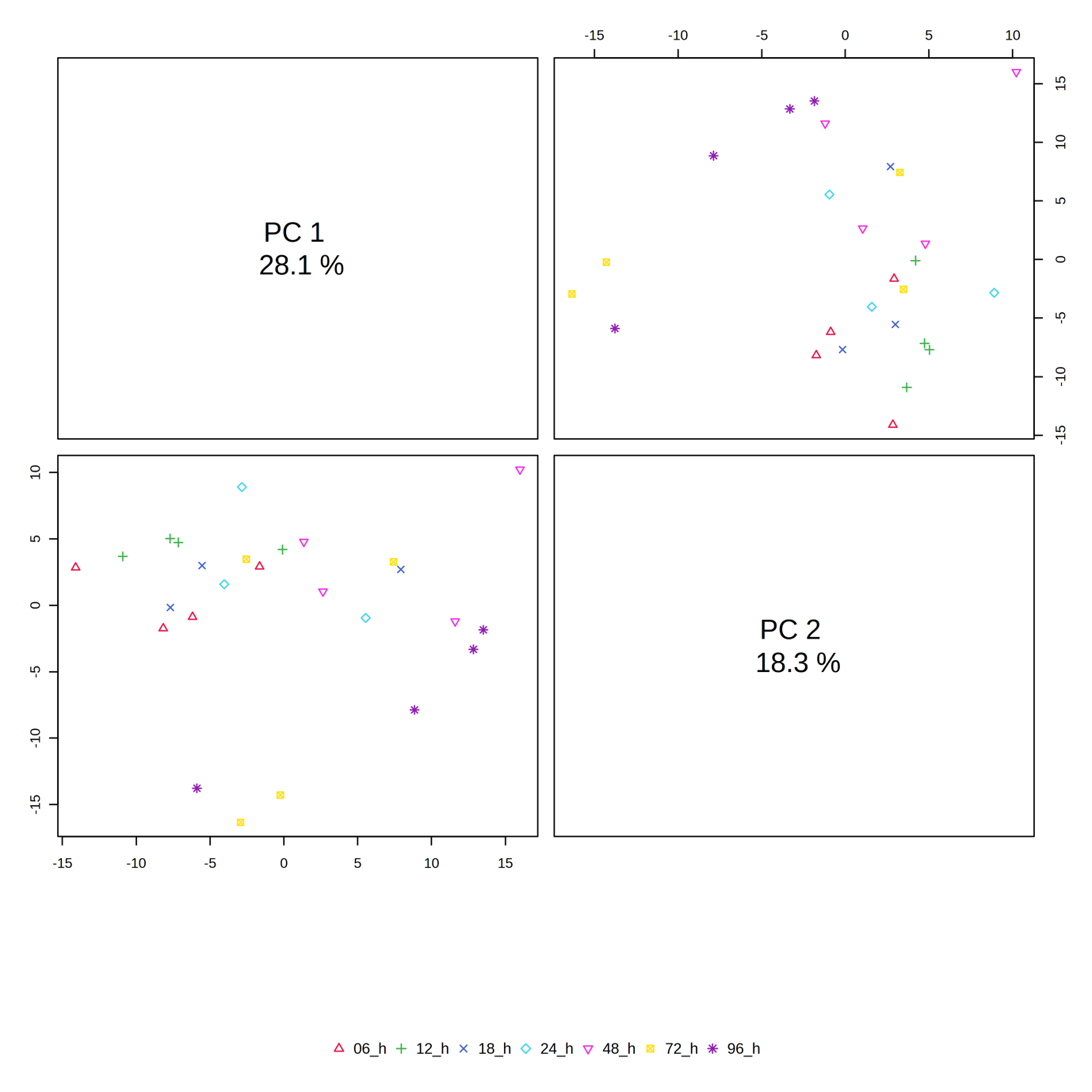


**Supplementary Figure 1:** Principal component analysis (PCA) for proteins that were ubiquitously quantified in all samples for either (A) kidney, (B) lung or (C) liver. No segregation of samples with different (short or prolonged) post-mortem times is apparent by PCA. Figures were generated using MetaboAnalyst (Pang Z, Zhou G, Ewald J, et al. Using MetaboAnalyst 5.0 for LC-HRMS spectra processing, multi-omics integration and covariate adjustment of global metabolomics data. *Nat Protoc*. 2022;17(8):1735-1761. doi:10.1038/s41596-022-00710-w)

# Supplementary Figure 2

| **A** | **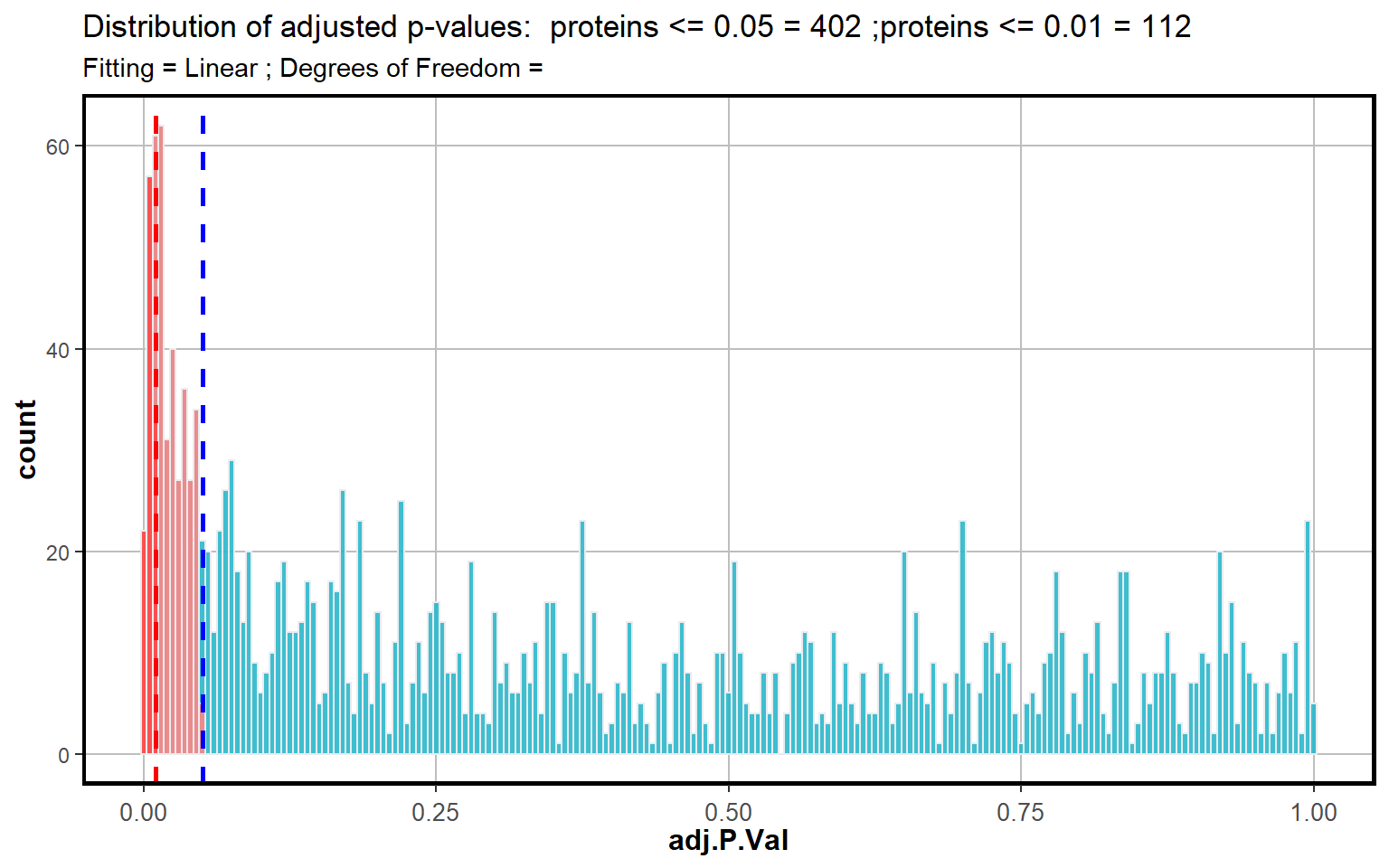** |
| --- | --- |
| **B** | **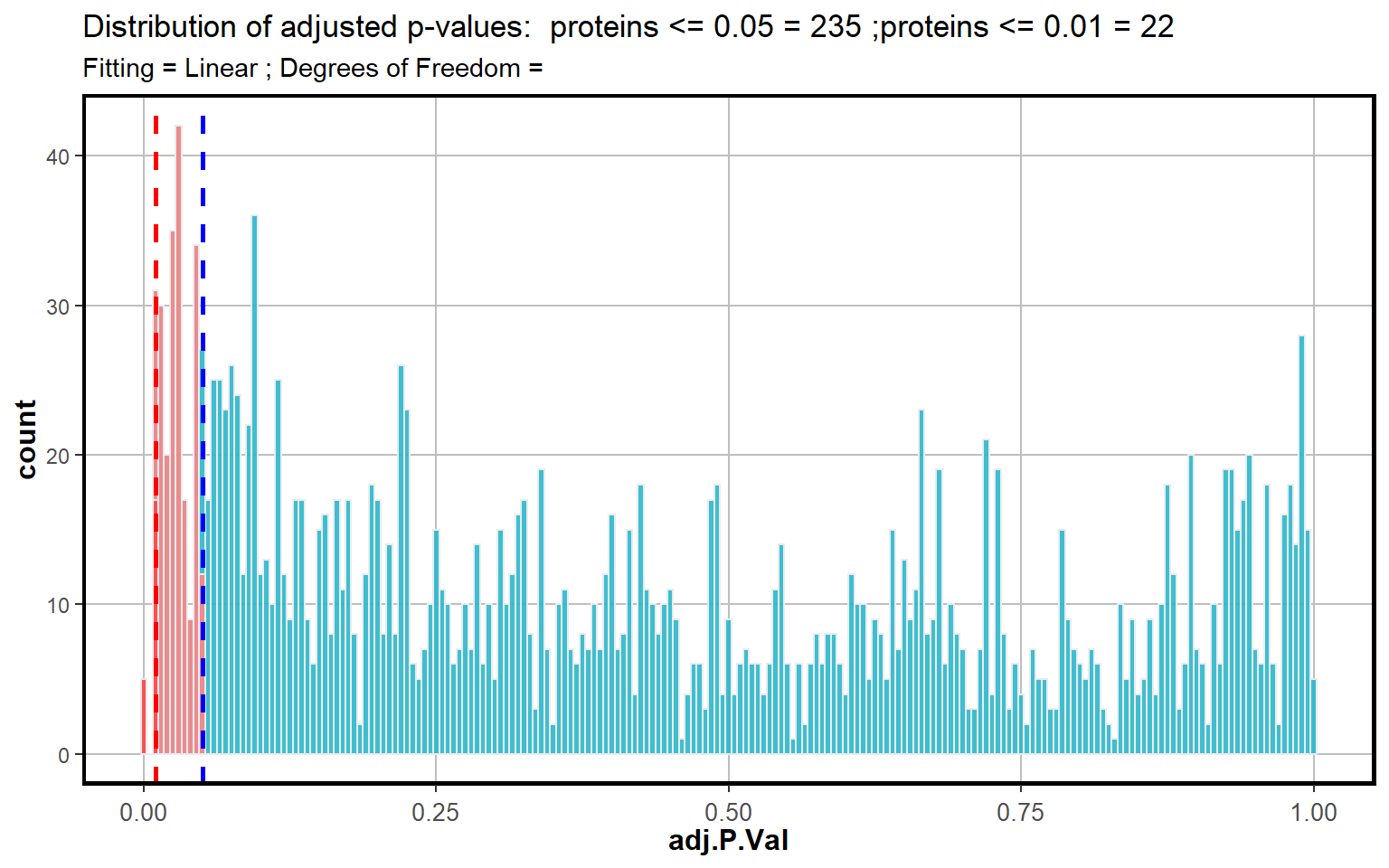** |
| **C** | **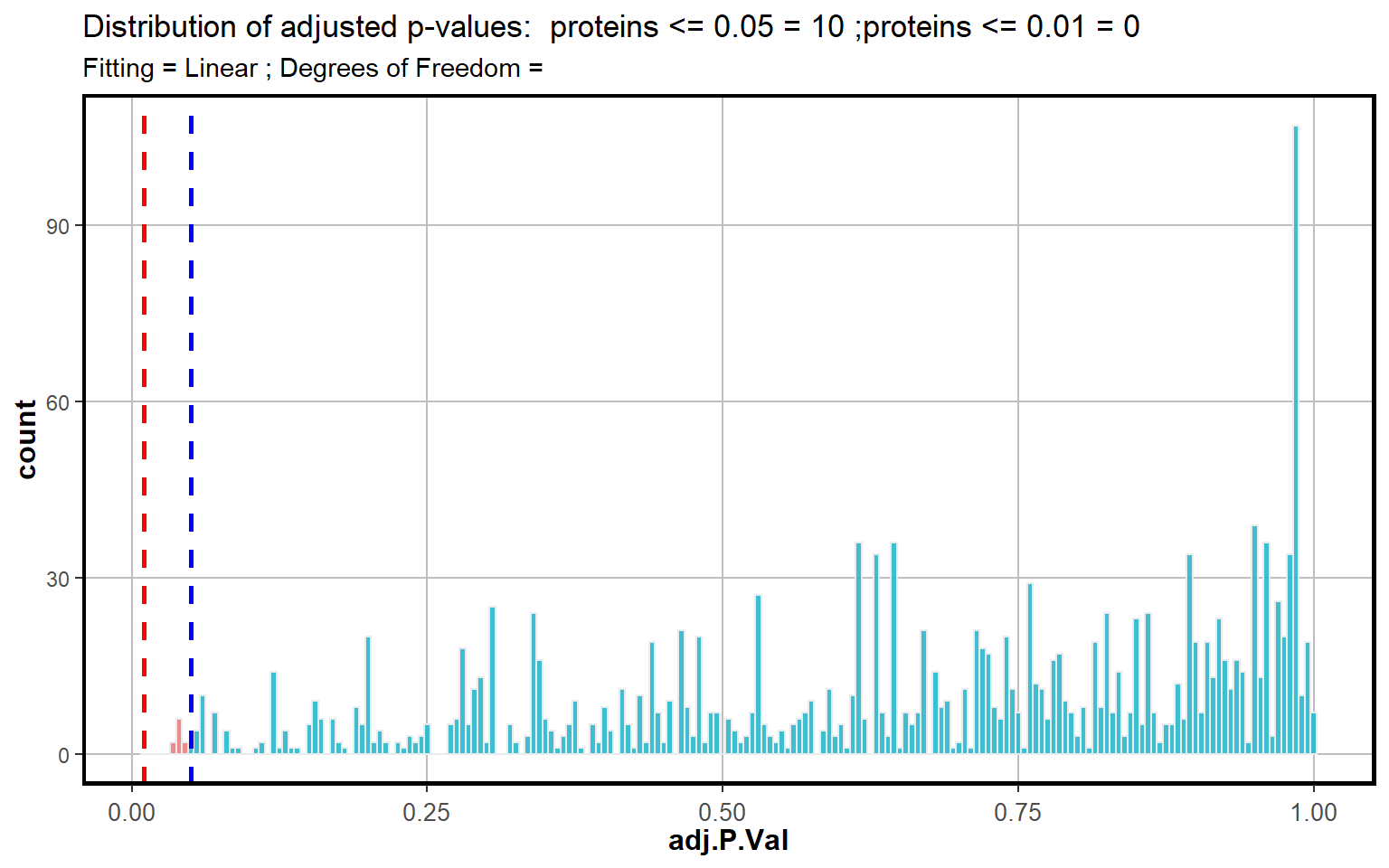** |
|  |  |

Supplementary Figure 2: Linear model with time as continuous variable for kidney (A), liver (B) and lung (C). The identified proteins have a distribution with an adjusted p-value ≤ 0.05.
